# ASEAN as network governance: An alternative lens to evaluate policymaking and performance

**DOI:** 10.12688/f1000research.136338.2

**Published:** 2023-10-02

**Authors:** Pushpanathan Sundram

**Affiliations:** 1 Chiang Mai University, Chiang Mai, Thailand

**Keywords:** ASEAN; network governance; policy networks; soft institutionalisation

## Abstract

**Background:** This article offers a new perspective of the Association of Southeast Asian Nations (ASEAN) as network governance (NG) using the policy network theory (PNT) for the analysis.

**Methods:** Two case studies were selected from the ASEAN Community to examine the applicability and effectiveness of NG in promoting ASEAN cooperation. These case studies were chosen due to their prominence and the prevalence of critical comments about their effectiveness in promoting cooperation. The content analysis utilised primary data from ASEAN reports, documents, research articles and processes, and studies on NG and policy networks. A literature review was conducted using online academic databases and ASEAN-related websites. The search covered the period from June 2019 to March 2021, with further searches conducted between January and July 2023. These data collection efforts provided a robust analysis of the case studies within the NG and ASEAN integration contexts.

**Results:** The findings show ASEAN as a hybrid structure which has supported its survival and relevance. This approach provides legitimacy for ASEAN globally by purposefully adopting structures similar to the EU without the supranational elements. It also showcases ASEAN’s motivation for asserting its centrality. Adopting a hybrid structure has led to ASEAN's soft institutionalisation, driven by its norms and characterised by a less empowered secretariat, where the member states are the decision-makers and implementors of policies. It opens a pathway to recognising ASEAN states’ autonomy, where the actors’ will and interests affect decision-making and define performance.

**Conclusions: **This article demonstrates NG's viability in promoting ASEAN's centrality in regional cooperation and institutional arrangements and allows for a nuanced assessment of its performance considering the context of the issue and ASEAN's history and emphasising the member states' primacy in policymaking. The NG framework contributes to ASEAN’s legitimacy, regional cooperation and performance despite its inherent limitations.

## Introduction

Despite the tremendous diversities, the Association of Southeast Asian Nations (ASEAN) has been primarily recognised for maintaining the peace and security of Southeast Asia (SEA) and promoting economic and sociocultural cooperation among its members. ASEAN attributes its success to the “adherence to the fundamental principles of non-interference and sovereignty with respect for shared values and norms” that have paved the path of tolerance for differences while highlighting the importance of keeping the region peaceful, secure, and stable ( ASEAN Secretariat, 2015). Notwithstanding this, the principle of non-interference and the lack of legally binding institutions in ASEAN underscore the ‘ASEAN Way’ and invite disapproval from critics and sceptics who regard it as an informal regional organisation incapable of achieving its goals and implementing its agenda in the absence of a central authority. Several scholars ( Leifer, 1999; Nischalke, 2002; Jones and Smith, 2006; Narine, 2009; Jones and Jenne, 2016; Emmerson, 2020) have pinpointed the inadequate formal institutions or mechanisms and legally binding agreements, soft institutionalisation, process-driven and normative structures led by weaker states as major shortcomings of ASEAN. As a result, ASEAN is often criticised for lacking meaningful progress in its regional integration prompting sceptics to propose that ASEAN adopt the more formal European Union (EU) institutions to perform better. These include a strengthened ASEAN Secretariat with legislative powers and enforcement authority like the European Commission and more robust dispute settlement mechanisms ( Ha *et al.,* 2016). In 2009, ASEAN embarked on building an ASEAN Community comprising the ASEAN Economic Community (AEC), ASEAN Political Security Community (APSC), and ASEAN Social Cultural Community (ASCC). It continues to develop the ASEAN Community through its blueprints for the three sectoral communities from 2016 to 2025. However, discrepancies observed between the “rhetorical goals and outcomes” of ASEAN integration or “implementation gaps” ( Jetschke, 2009) have underscored the Association’s norms and principles as shortcomings in its community-building agenda. While the adoption of central institutions and a legal framework to ‘force’ member states to comply and be accountable for their decisions has been a subject of dispute as a means to strengthen ASEAN’s institutions ( Koh *et al.,* 2009), it should be recalled that ASEAN’s current institutional design is but a deliberated and purposeful choice of the founders of ASEAN without which we would not have seen the survival of ASEAN. As such, these perceived weaknesses within ASEAN institutions impacted much of the Association’s history, as well as the work that has gone into the policymaking process in the pursuit of ASEAN goals while balancing member states’ interests. Therefore, it will be important to recognise the embedment of norms and principles in ASEAN policies and agreements. Yet, it does not necessarily impede or obstruct ASEAN goals and policy implementation in issue areas where member states’ interests precede that of the underlying norms and principles of ASEAN.

Accordingly, the article proposes an alternative framework of network governance (NG) to provide a nuanced account of policymaking, taking into account the networked institutional design of ASEAN using the policy network theory (PNT) of Michael Howlett (2002). Here, we can acknowledge the progress and performance of ASEAN via the PNT, which assesses ASEAN’s policy processes and outcomes through the interaction between the role of actors, ideas, and interests in the policymaking process. This makes way for the inclusion and consideration of ASEAN norms and principles (ideas) and how they intersect with member states’ will (actors) and ASEAN goals (interests). This context of policymaking considers ASEAN’s norms and principles (underpinnings) and will assist in evaluating the Association without prejudice to these underpinnings. Utilising PNT, this article will present case studies from the AEC and APSC to evaluate the performance of the communities, going beyond identifying the Association’s shared norms, such as the principle of non-intervention and sovereignty, as the cause for implementation failure or a lack of performance. In particular, by focusing on the interaction between actors, ideas, and interests, attributing more responsibility to member states in strengthening implementation gaps and enhancing performance and thereby recognising that the ‘non-institutionalised form of regionalism’ of ASEAN ( Acharya, 2008) can bring performance and non-performance.

### Literature review

Established in August 1967 by Indonesia, Malaysia, the Philippines, Singapore, and Thailand as the five founding members of ASEAN, they held the vision of promoting and maintaining security and peace in the SEA region. Against the backdrop of the Cold War, most SEA states found themselves “threatened by subversive communist movements” ( Kefale, 2015) in addition to a series of territorial and border disputes. The latter led to the task of state consolidation for the founding members during the development of ASEAN for fear of neighbouring conflicts and history from their colonial past. It was clear that distrust between SEA states ran deep during those times, especially with Konfrontasi (or Confrontation) pursued by then-Indonesian President Sukarno between 1963 and 1966. It was ultimately a policy which opposed the formation of the Federation of Malaysia since it was viewed as a “British imperial project” ( Kefale, 2015). Konfrontasi paints a clear picture of the then-faltering dynamics between Indonesia and other SEA states and Indonesia’s ambition to project its foreign policy goals abroad through armed conflicts, which bred scepticism and potentially undermined regional cooperation and multilateral ties among regional states. Nonetheless, ASEAN still managed to emerge from these complex dynamics, with its main objectives focused on mitigating regional threats and maintaining regional security.

History has shown that the norms of engagement in ASEAN would take a different trajectory from other regional organisations like the EU, considering the colonial occupation that most ASEAN member states have been through. For instance, it would be difficult to reconcile the idea of ‘pooling sovereignty’ as seen in the EU and even delegate some legal mandate to an extra-national body like ASEAN when sovereignty is a hard-earned freedom in the nation states of SEA. Only by being able to exercise a traditional understanding of sovereignty and non-interference as a regional principle ( Chetchaiwong, n.d.) do member states ensure that domestic issues remain an internal affair of the relevant member states, safeguarding their right towards conflict management. While this may reflect a realist position and may be incompatible with policymaking in a regional organisation, it provided autonomy and safeguarded the principle of non-interference sacrosanct to all ASEAN member states, thereby allowing for cooperation and, in the later years, regional integration. The ASEAN founders, when establishing the Association in 1967, had based its principles and norms on ‘musyawarah’ (consultation) and ‘mufakat’ (consensus), which characterise what many have termed as a ‘loose and informal’ cooperation style.

To recommend ASEAN undergo institutional evolution to emulate the successes of the European integration project proposes an approach which lacks an understanding of ASEAN and the rationale behind the principles and norms. As such, it would be important to recall some critical components that critics have conjectured as reasons that resulted in the gaps between ASEAN’s rhetoric goals and its actual performance or implementation gaps. The reliance on socially rather than legally binding rules and norms and its lack of a centralised and autonomous secretariat ( Jetschke, 2009) highlights ASEAN’s light form of institutionalisation, which in turn produces network forms of governance ( Jetschke, 2009) encapsulated by the concept of NG reasoned to be behind ASEAN’s implementation gaps due to the inability to command compliance from its member states. In Jetschke’s argument, the concept of NG reiterates member states’ high regard for their autonomy and their unyielding attitude toward the principle of non-interference and a weak manner of institutionalisation that affects the unanimity on policy issues, decision outcomes, and the rate of implementation or adoption of a policy in ASEAN. However, this article views NG as a viable alternative theoretical framework which can better account for ASEAN’s performance and non-performance without prejudice to its norms and principles.

NG has more prominence in its own field of study in the literature on policy networks. First, networks indicate non-hierarchical and knowledge-based forms of policymaking that enable the progression of ideas, communication, and consensus-building processes for specific policies ( Mayntz, 1993; Rhodes, 2003). These characteristics are aligned with the policy networks of ASEAN that focus on networks as communication devices. At the fundamental level, the policy network theory (PNT) proposes a framework detailing how different subsystem setups relate to specific paradigmatic processes of policy change, suggesting that some are linked to propensities for particular types of policy change ( Howlett, 2002). Policy networks are also interdependent as networks are mutually reliant on each other’s resources ( Bevir, 2011) and often referred to as “mutual resource dependence” ( Bevir, 2009). Klijn (1997) suggests that interdependencies engender interactions between actors and sustain relation patterns in policy networks. Hence, policy network theory or the ‘network approach’ is pivotal in examining ASEAN as a ‘network structure’ and the effectiveness of the numerous types of networks contained in the Association. This approach deconstructs how we view the organisation’s structure, policy process, and outcomes.

For Jetschke (2009), “if one subtracts Europe’s effects on the organisation, ASEAN’s formalisation since 1967 might well amount to pure NG”. Hence, the maintenance of autonomy, strong regard for the principle of non-interference, and the Westphalian notion of sovereignty are obstacles to the development of an EU-like institutional design in ASEAN, reinforcing the preference for an informal policy style and a non-binding set of rules and principles ( Jetschke, 2009). Yet, without the agreement to those norms and principles, the establishment of ASEAN would not have been possible. Since these norms are practically non-negotiable at this juncture, developing a framework to reflect these underpinnings is paramount. Therefore, the article seeks to utilise the concept of NG to conceive of ASEAN’s institutional design but concludes on a different note from Jetschke by highlighting the potential that NG holds in evaluating and nurturing the potential of ASEAN.

First, NG is an apt framework for ASEAN because it is “an alternative to hierarchy and a viable approach for theorising questions of order while not downplaying the extent of egoism among ASEAN members” ( Jetschke, 2009). Where networks are “lighter on their feet than hierarchies”, and exchanges are carried out only by the members of the network(s) through “reciprocal, preferential, mutually supportive actions” ( Powell *et al.*, 1990). A similar definition was also proposed in the work of Jones, Hesterly, and Borgati, which views NG as a select, persistent, and structured set of autonomous organisations engaged in non-legally binding contracts to adapt to environmental contingencies and to coordinate and safeguard exchanges ( Jones *et al.,* 1997). Thus, NG presents itself as a useful framework with its emphasis on the importance and precedence of states (states’ autonomy) and the exchanges between member states that define the networks forged in the organisation itself ( Kim, 2006). In this regard, it reflects the intergovernmental nature of ASEAN but does not point to an absence of institutional structure. It gives room to consider an alternative to hierarchy. Moreover, it suggests that ASEAN’s light institutionalisation is not a compromised design or a ‘middle ground’.

It would be noteworthy to consider how Powell indicated that in a network organisation, the method of conflict resolution would be heavily dependent on the ’norm of reciprocity agreed among members’ and, as such, subject members to an interdependent relationship ( Powell *et al.,* 1990). It means that the concept of NG would view the overarching norms of engagement and/or means of conflict management as a key essence of the organisation’s core. This would indicate NG’s capacity to view ASEAN’s norms and principles as essential makings to the character and performance of ASEAN. Highlighting the norm of reciprocity also suggests member states take lead roles when engaging or enacting specific policies depicting that power lies with them ( Coen and Thatcher, 2008). Finally, it validates ASEAN’s current functioning by emphasising ’consultation, negotiation and soft law’ ( Coen and Thatcher, 2008).

Using the NG framework, the article views ASEAN’s norms and principles as inevitable and permanent components defining the norms of engagement and degree of institutionalisation in the Association. It highlights the interplay between the former and the role of member states, which will define how it manages and mitigates challenges. In addition, it allows us to view member states as conscious and active actors responsible and accountable for the interplay we witness or observe during policymaking. As Powell signalled, a key to network forms of organisation is that “expectations (within the organisation) are not frozen but change as circumstance dictate” ( Powell *et al.,* 1990). Here, member states hold the authority and power to make changes. Hence, as Powell stipulates, complementarity and accommodation are pertinent features to hone for a network organisation to succeed and where the article holds ASEAN’s challenges to be as we observe if the will or interests of member states precedes that of the maintenance of ASEAN’s norms and principles especially in the political and security issue-areas. While the Association could find itself in a deadlock as the policies in place are not backed up by legally binding rules and regulations ( Stubbs, 2019), NG can help to identify the stage or phase at which a deadlock occurs, that could further hone its success in areas of policy formulation, coordination, and implementation. As such, the light institutionalisation of ASEAN would be better represented in a nuanced manner through the concept of policy networks supported by NG.

Given the central role of member states’ beliefs and perceptions in shaping ASEAN policymaking, it may appear redundant for sceptics to question granting states a high degree of autonomy within a regional organisation. The fact that sceptics, realists, and neo-realists often believe ASEAN to be an organisation in which “style trumps substance” and an organisation in which process, especially the ASEAN Way, is “emphasised over progress” ( Stubbs, 2019) stresses how inefficient ASEAN is perceived in their view. Yet, to expect a change of the ASEAN Way to a more legalistic approach, less focused on the values, beliefs, and norms imbued since the beginning and defined by colonial past and history for a more effective organisation could just be ideal. From a divisive region (during the Cold War era) to a region that can now boast about more amicable diplomatic ties among member states, the importance of identity, values, and norms is not stressed sufficiently. The member states’ preferences and interests should be acknowledged as key to ASEAN’s performance in shaping policy outcomes.

A brief comparison between the economic and political realms to showcase the degree of the interests of the member states would illustrate the above. In matters of economic pursuits, ASEAN member states are keen to pursue and establish free trade areas (FTAs) and the lowering of tariffs and non-tariff barriers. It is evident in the ASEAN Free Trade Area (AFTA) agreement signed in 1992, the ASEAN Trade in Goods Agreement (ATIGA) in 2009, and ASEAN member states’ interest and willingness to be a part of the Regional Comprehensive Economic Partnership (RCEP) concluded in 2020. On the other hand, issues in the political realm tend to be at a standstill, with member states seemingly ‘handicapped’ despite an expressed interest in resolving them due to the ASEAN principles and norms. For example, the territorial dispute in the South China Sea (SCS) has stretched over 20 years, with consultations in place to establish a code of conduct in the SCS, but to date, it has not been successful. As such, the will of member states is the critical variable affecting the cooperation and performance of ASEAN. Cooperation ensues when the interests and will converge, and when they diverge, non-performance occurs depending on the norms and principles impacted.

With this, an alternative theoretical framework of NG will be utilised to capture a more nuanced understanding of the underpinnings of ASEAN while focusing on the role of member states as active actors capable of shaping decisions and wielding the authority to act beyond what the norms and principles of ASEAN would dictate in issue-areas where the will or interests of member states take precedence.

### Analytical framework

In establishing the framework of NG first and foremost, the article adopts a specific definition of the framework provided by Ostrom, which identifies it as “a set of general variables and relationships that could be studied to understand a particular phenomenon but assigns no values to the variables and does not specify the direction of relationships between them” ( Araral *et al.,* 2015). A framework is essential to capture the essence of ASEAN and identify the interactions between actors that dominate and participate in the policy processes of ASEAN. It would also be supported by Michael Howlett’s PNT, which gives impetus to “thinking about policy making as involving more-or-less fluid sets of state and societal actors linked together by specific interest” ( Howlett, 2002). The article uses the PNT approach to organise “actors and institutions into identifiable sets of policy-relevant interactions” ( Howlett, 2002). It looks beyond structure (institutional mode of analysis) or agency (behavioural mode of analysis) when assessing a policymaking process. This offers a fresh perspective on the role of actors and interests and evaluating policy outcomes – whether they are/were successful or failed outcomes.

Utilising the concept of a policy universe which “can be thought of as an all-encompassing aggregation of all possible state, private and social actors at various levels (local, regional, national, international) working within the institutions that directly or indirectly affect a specific policy area” ( Howlett *et al.,* 2017), ASEAN is conceptualised as a policy universe. The idea of a policy universe is a nuanced manner of conceptualising the institutional arrangement of ASEAN. It differs in terms of organisational design, where the latter is directly concerned with the institutions and degree of formalisation, while the former goes beyond the notion of institutional design as it looks at how the relationships and dynamics between member states and the actors and stakeholders outside of ASEAN who may be involved in the policymaking process. This could affect the agenda and outcome of the organisation and thereby impact the performance or non-performance of the Association.

If we recall how ASEAN founders established ASEAN for the purpose that “had to do mainly with regional peace and security” ( Severino, 2003), it becomes clear that this rationale will impact the performance of ASEAN. This means that ASEAN’s ability to overcome or adapt to changes in the geopolitical landscape within the region shaped by great power, regional interests and transnational issues will be the points of assessment. In this regard, managing its centrality in regional geo-political arrangements and socialising or influencing decisions aligned with ASEAN’s views are geopolitical considerations rather than pursuing through materialistic means. Thus, ASEAN’s performance will be related to how it safeguards its relevance in any arrangement or drives its centrality.

Here, ASEAN’s centrality relates to how ASEAN is able to assert its shared norms and values as an organisation and one of the essential roles of ASEAN is to manage its centrality in regional geo-political arrangements. Where ASEAN’s centrality does not simply refer to its identity in the region, it stresses and signals the capacity, in terms of its influence and power, the organisation wields in the region. It would be apt to refer to how “centrality is seen to indicate the social power of an actor based on how extensively it is connected to the entire network” ( Caballero-Anthony, 2014). The more ASEAN can assert itself, the better it can drive centrality in the networks it belongs to. To further make sense of the gravity of ASEAN’s centrality to the organisation, the article turns to the Resource Dependence Theory (RDT) to underscore how the relevance and the continued survival of the Association can be argued to be as critically connected to the survival of the Association’s member states. From an RDT’s perspective, it refers to the (regional) organisation as a ‘critical resource’ that members belonging to that regional organisation depend upon, and thus, understood as “important resource providers” ( Drees and Heugens, 2013). As such, to a large extent, driving ASEAN’s centrality is an important task of the organisation. Since ASEAN’s survival is dependent on how it engages with its cluster of networks to facilitate interdependencies and solidify its legitimacy ( Drees and Heugens, 2013), it becomes all the more crucial to conceive of the dynamics and relationships in ASEAN as a policy universe to pinpoint member states exercise of leadership towards consolidating ASEAN goals in an intergovernmental organisation setting. As such, centrality provides another expression of ASEAN’s power in a constructivist sense ( Caballero-Anthony, 2014), where we view how capable member states are at driving ASEAN’s centrality in pursuing its goal and international legitimacy.

On the other hand, a policy subsystem defines as “the actors active in each sector or issue area” and/or are “forms of networks which encompass the interrelationships existing between elements of the policy universe active in specific knowledge and political spaces” ( Howlett *et al.,* 2017) This article views the three pillars of the ASEAN Community: ASEAN Economic Community (AEC), ASEAN Political Security Community (APSC), and ASEAN Socio Cultural Community (ASCC) as policy subsystem where each is unique with varying degree of significance to different member states including actors’ bargaining ability would define the performance or policy outcomes of the community. In utilising the concept of NG, the interactions between actors in the policy network are critical to understanding the interplay between various actors’ will and interests.

## Methods

A qualitative content analysis and case studies related to the significance of NG and policy networks in ASEAN were undertaken. Through qualitative content analysis, relevant information was systematically analysed to identify patterns and themes. The case studies provided specific examples that shed light on the dynamics of policy networks, member states’ motivations, and the role of ASEAN’s centrality in policymaking. Combining these methods, the study aimed to understand the relevance and application of NG and policy networks within ASEAN.

### Content analysis

The primary data used for the content analysis was ASEAN reports, documents, and research articles, besides analysis of studies and research papers on NG, policy networks, and ASEAN processes. They provided an understanding of the policy process phases and their varying degrees of success in the individual ASEAN communities.

This study’s search strategy for documents and sources involved a comprehensive approach. A systematic literature review was conducted using online academic databases such as Google Scholar, ResearchGate, Academia.edu, and ASEAN Secretariat and ASEAN member states websites. The search terms included the following keywords: ASEAN, ASEAN Economic Community, ASEAN Political Security Community, ASEAN Secretariat, AFTA, ASW, ARF, AMMTC, network governance, hybrid organisations, policy network, resource-dependence, state actors, non-state actors, soft institutionalisation, norms, non-interference, sovereignty, EU network governance, and EU policy networks. They were searched in relation to network governance, policy networks and policymaking and effectiveness. The words were primarily identified prior to the analysis. They were categorised and classified under several headings for systematic comparisons and analysis: EU and network governance; ASEAN and network governance; ASEAN network structures; EU network structures, state and non-state actors in ASEAN; norms, soft institutionalisation and hybrid organisation; and ASEAN motives, policy-making and performance in AFTA, ASW, ARF and AMMTC. The search was conducted between June 2019 and March 2021, covering a wide range of scholarly articles, books, reports, and policy documents as part of the author’s doctoral research. The author conducted further searches between January and July 2023 in developing this article.

Data collation involved a two-step process. First, relevant literature was gathered through the systematic literature review. The selected documents were then subjected to content analysis using the categories identified and mapped to compare and contrast the case studies and the differences between ASEAN and EU network governance. The variables included in the analysis focused on understanding the structure, functioning, and effectiveness of ASEAN as a hybrid organisation with network governance at its core. These variables encompassed aspects such as organisational legitimacy, decision-making processes, policy implementation, and the role of member states in shaping ASEAN’s performance.

The methods of analysis employed in this study were qualitative. Content analysis was used to examine the gathered literature and identify recurring themes, patterns, and theoretical frameworks related to network governance and hybrid organisations. The findings from the literature analysis were synthesised and interpreted to provide a comprehensive understanding of ASEAN’s hybrid structure and its implications for the organisation’s legitimacy and pursuit of centrality.

### Case studies

Two case studies, one from AEC and another from APSC covering two policy networks each, were utilised to study the presence and workings of NG in ASEAN alongside the resource dependence among member states that impacted policymaking.

**Figure 1.**
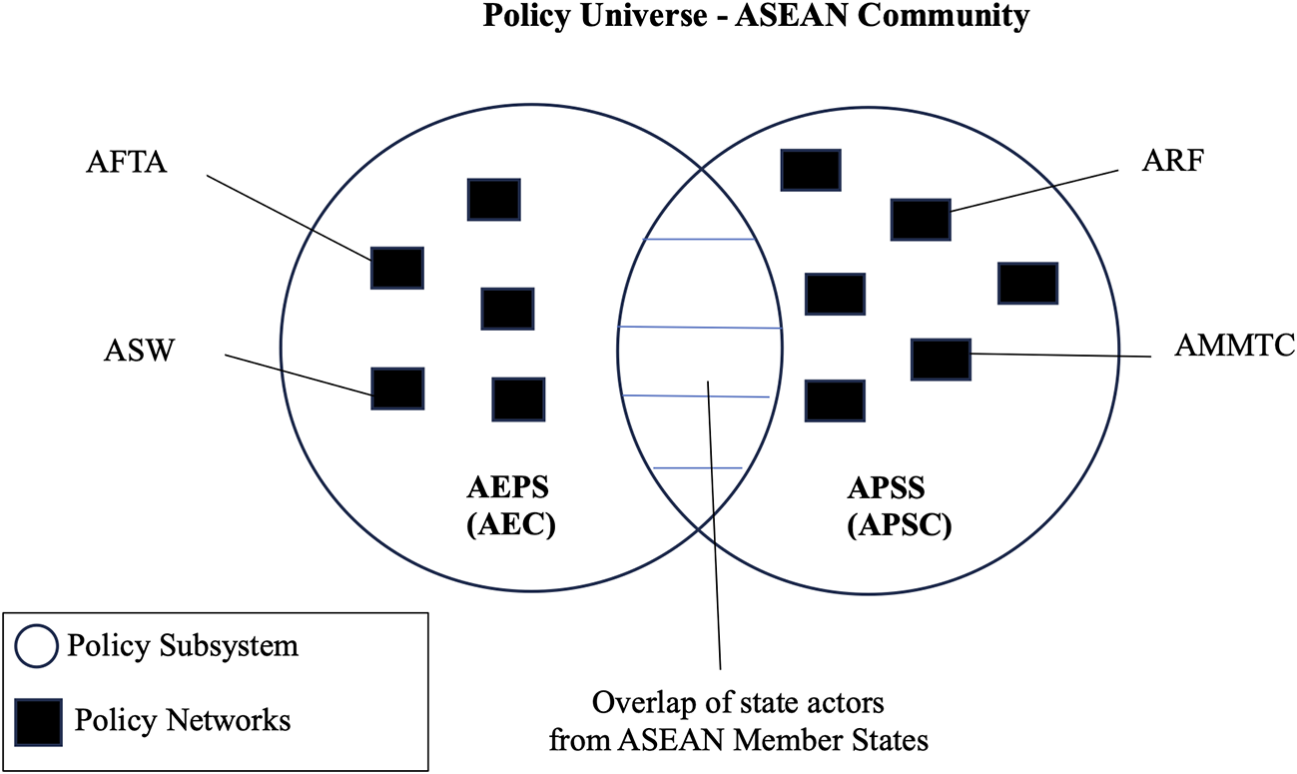
Network Governance and ASEAN.

The selection of AFTA as a case study within the ASEAN Economic Policy Subsystem (AEPS) is a deliberate choice, as the AFTA was established before the AEC itself. Moreover, with its long and substantive history, AFTA is a significant policy network to examine the performance of AEPS. Its significance is pertinent because AFTA’s framework-agreement approach was used to launch negotiations for the AEC and establish other regional free trade arrangements. Besides, the ASEAN Single Window (ASW), a core component of AFTA, was selected as it is a relevant micro case study on the operations of NG in AFTA, where custom cooperation and trade facilitation are forged across the region. Likewise, the ARF was identified as a case study since it is recognised as a “venue for multilateral and bilateral dialogue and consultations and the establishment of effective principles for dialogue and cooperation” (ASEAN Secretariat, n.d.-b), where a myriad of political and security issues is discussed. On the other hand, the ASEAN Ministerial Meeting on Transnational Crime (AMMTC) policy network was chosen as a contrast to ARF, where the focus on non-traditional security has often relied on the partnerships and cooperation of non-state actors (NSAs), which points to some acceptance of them in the APSS and at the discretion of member states. Figure 1 depicts the policy universe of the ASEAN Community with the two communities (AEC and APSC) and the policy networks (AFTA, ASW, ARF and AMMTC).

## Results

### Case study one: ASEAN economic policy subsystem – AFTA and ASW

Theories of regional economic integration would see the formation of the AFTA as one that encourages trade creation within the ASEAN bloc and would propel intra-regional trade flows and boost member states’ economies. It would result in greater integration as member states’ economies become increasingly interdependent. As such, the performance or non-performance of the economic integration is assessed through quantitative indicators such as changes in the share of intra-regional trade and investment since these are closely involved with indicators of interdependence. However, when these traditional quantitative indicators are applied to ASEAN, it paints a disappointing outlook since trade creation was highlighted as a main reason for entering regional economic arrangement, yet the share of intra-regional trade remained low at 20% or less ( Bowles, 1997).

Hence, mainstream theories of regional economic integration coupled with traditional metrics tend to reduce ASEAN’s ambitions and objectives to a linear wealth creation project. Bowles argued that the role of AFTA was to bring “global capital to the ASEAN region” by adopting liberalisation in ASEAN and moving towards a “rules-based, fully liberalised global economic system” ( Stubbs, 2019). Through Bowles, we understand that the establishment of AFTA by ASEAN is an exemplification of open regionalism and a characteristic of the new regionalism, allowing members of AFTA to actively participate in the global economy and receive economic advantages by establishing economic benefits ties within a global network. When the role of AFTA is understood as such, an innocent inspection will highlight the desire of ASEAN to be a part of the “liberalised global economic order” as well as AFTA as a “stepping-stone” to reaching it ( Stubbs, 2019). Yet this narrative places AFTA in a tricky position in suggesting that its primary purpose is to increase regional integration outcomes ( Menon, 2021), thereby inviting scrutiny from a perspective lacking an understanding of the ASEAN context. While AFTA may be perceived as an initiative encouraged by new regionalism ( Stubbs, 2019), the supposed benefits of participating in the global economy subsume what should be achieved for the AEC to be deemed successful.

From the policy network perspective, it is known that ASEAN member states agreed to establish the AFTA policy network even though agreeing to participate in FTA signals the loss of some sovereignty as member states’ economies become closely tied. Therefore, the interests that once moved the policymaking process can be understood via RDT, where the Association is a ’critical resource’ for member states as an organisation made up of new states. As such, member states would not only harbour the goal of economic growth but also pursue economic viability as a region to become economically competitive compared to other regions to boost its centrality by demonstrating its relevance to the international society by joining the global economic order. Therefore, where ASEAN is a critical resource to its member states, and member states are to the Association, the policy network of AFTA becomes all the more pertinent and connected to each other’s survival as stipulated by RDT.

Here, it would also be constructive to consider what Menon (2021) has articulated, where “an outsider observing these outcomes would have declared the program (AFTA) a failure while an insider would have understood that they achieved exactly what they were designed to achieve: very little” ( Menon, 2021). This goes back to the main issue where “low shares of intra-ASEAN trade have attracted a lot of critical comment, and some have used it to question ASEAN’s viability as a regional group” ( Hill and Menon, 2011), going further to suggest that this indicates non-performance. However, the crux of the issue lies with a superficial interpretation of intra-ASEAN trade shares. Where it would be essential to bear in mind that “the ASEAN economies only account for a small share of global trade flows”, thus “when adjusted for this scale factor—through the computation of trade intensity measures for instance—the picture that emerges is quite different” ( Hill and Menon, 2011).

In addition, it would be essential to understand the rationale of the program based on ASEAN’s role and needs where since “AFTA trade concessions are multilateralised, the observed increase in intra-regional trade shares must be explained by complementarities and market-driven factors rather than deliberate policy measures” ( Hill and Menon, 2011). For instance, in the case of “both Singapore and Malaysia, ASEAN markets constitute more than one-quarter of total exports. The “share is much lower for Indonesia, where natural resource exports to extra-regional markets are important, and for the Philippines, whose commercial patterns have always been the least ASEAN-centred of the five original member countries” ( Hill and Menon, 2011). This helps to elaborate how economic integration served ASEAN both in terms of the pursuit of globalisation and one that pertains to the continued relevance and survival of the Association and its very own centrality in the face of an international system.

Given this, it would be fatal to conflate the goal of pursuing regional resilience with that of an inward-focused task unreceptive to open regionalism ( Stubbs, 2019). It differs because we approach it from the understanding of centrality, where we view member states’ motivation to push through the AFTA policy network as a result of resource dependency – a crucial element of NG, where it becomes a significant variable influencing member states’ involvement since member states depend on ASEAN and ASEAN relies on its members to maintain relevance in the global economy, the international system, and its survival.

Due to the economic benefits and to facilitate intra-ASEAN trade liberalisation and facilitation, the member states have worked towards the integration of customs procedures, documentation and information exchange systems, reducing paperwork, duplication, and processing time through the ASW, thereby saving business costs, streamlining trade processes and improving efficiency in cross border trade. By providing a single electronic gateway for customs-related processes and documentation, the ASW reduces administrative burdens, enhances transparency, and expedites the clearance of goods at borders ( Sithanonxay and Neo, 2022). This strengthens intra-ASEAN trade, and the trickle-down effect will be the increased trade flows, accounting for a quarter of ASEAN’s total trade. It also makes the region competitive and attracts trade and investment from its free trade agreement partners, such as China, Japan, South Korea, India, Australia and New Zealand. In fact, member states such as Singapore and Malaysia, which see a benefit from intra-ASEAN trade, have accelerated the adoption of the ASW. In contrast, those member states which see lesser benefits have been slower to take up. In addition, state actors such as customs authorities and relevant ministries collaborated within each member state and within a network governance framework to harmonise policies and regulations and enhance coordination to enhance intra-ASEAN trade facilitation.

With this, ASEAN’s move towards AFTA has more to do with its self-preservation as a leading regional entity in East Asia and to buffer against external economic shocks. Creating FTA between member states for economic benefits establishes a footing for all ASEAN member states, especially since ASEAN comprises developing and smaller economies than other regions. This also moves away from the rationale that the AEC is a relatively more successful policy subsystem due to pure economic gains from the global economy. However, it highlights member states’ focus on regional resilience, which, in this case, is the ability to withstand financial crises or external political threats. After all, an organisation emphasising the principle of sovereignty and non-interference naturally articulates the preference to adhere to a self-help mentality, and purely economic gains could not be sufficient for AFTA’s establishment.

### Case study two: ASEAN Political Security Policy Subsystem – ARF and AMMTC

As part of the ASEAN Community, the ASEAN Political Security Policy System (APSS) oversees political and security cooperation to preserve and safeguard the ASEAN region’s peace and security, encompassing the ARF inaugurated in 1994. The objectives of ARF are to foster constructive dialogue and consultation on political and security issues of common interest and concerns and make significant contributions to efforts towards confidence building and preventive diplomacy in the Asia-Pacific region ( ASEAN Secretariat, n.d.). The participants of ARF are the ASEAN member states and Australia, Bangladesh, Canada, China, India, the EU, Japan, Mongolia, New Zealand, North Korea, Pakistan, Papua New Guinea, Russia, South Korea, Sri Lanka, Timor Leste, and the US. This set of fixed members in the ARF network hold repeated interactions with each other over time through annual ministerial meetings, aligns with the concept of NG as involving a “select, persistent, and structured set of autonomous entities [firms] based on implicit contracts to adapt to environmental contingencies and to coordinate and safeguard exchanges” ( Jones *et al.,* 1997).

By encapsulating the interaction of these stakeholders in a policy network, NG helps us to approach the idea of a structural position where each stakeholder is understood as a “node” ( Caballero-Anthony, 2014). Where member states’ drive, interest, and willingness to participate from the standpoint of policy network analysis are linked to the RDT as they understand the importance of the Association to their own survival, it would suggest the pursuit of interaction within the network from member states to a stakeholder or partner within ARF, prompting a high level of actors-to-actors (node-to-node) interactions ( Caballero-Anthony, 2014) to keep this network relevant. This is where the notion of centrality is invoked again, as seen in the AFTA under AEC, where regional resilience is pursued through participation in the global economy and in the case of the ARF, ASEAN’s attempt to configure its relevance by probing itself as a significant “bridging player” ( Caballero-Anthony, 2014) by initiating and convening the regional forum. It is significant as it expresses how ASEAN achieves its goal via the ARF by acting as a middle platform for world leaders to congregate and provide opportunities in two areas. One, dialogue partners to meet others to promote their interests; and two, dialogue partners to extend, promote or strengthen their presence in the Asia Pacific region, including with ASEAN. The Association prides itself and strives to continue playing its role as an honest broker facilitating the ARF network, which adds to ASEAN being relevant to the security and peace of Asia Pacific. Yet, while this may be aligned with ASEAN goals, the ARF can be bounded by ASEAN’s norms and principles, limiting it to only mere deliberation over security and political issues since it is highly dependent on how member states pursue ’node-to-node’ interaction.

Regarding AMMTC, ASEAN’s development of a coordinated approach stemmed from the abuse of narcotics and trafficking in illegal drugs. On 24 February 1976, ASEAN member states signed the Declaration of ASEAN Concord, tightening the resolve to fight transnational crime and intensifying cooperation among member states, including relevant international bodies, in the prevention and eradication of the abuse of narcotics and the illegal trafficking of drugs ( Sundram, 1999; Parameswaran, 2000). ASEAN resolved to take firm and stern measures to combat transnational crimes such as drug trafficking, trafficking in women and children, and other transnational crime at their 2nd Informal ASEAN Summit in 1997 by adopting the ASEAN Vision 2020 document whose main objective is the creation of a drug-free Southeast Asia and a region of agreed rules of behaviour and cooperative measures to deal with problems that can only be met on a regional scale ( Parameswaran, 2000).

This shows that transnational crime remains a significant concern for the Association, especially with the diversification of transnational crime to include terrorism, arms smuggling, money laundering, illegal migration, and piracy, and more notably, cyber security crimes, which are becoming highly organised in nature ( Sundram, 1999). Moreover, the transnational crime policy networks involved cooperation from many areas, such as law enforcement, regional and international organisations, and local authorities’ cooperation. Transnational crime policy networks rely upon both ASEAN member states and NSAs and, as such, can be understood as inclusive policy networks where the roles of any parties within the policy network are not undermined. It returns to the concept of RDT, which views the Association as an essential critical resource for members. The transnational nature of the policy network required ASEAN member states to rely on local authorities and NSAs. As explained by Howlett, the dynamic nature of such interaction among different actors fosters a healthy, balanced policy network that would produce dynamic policy outcomes.

The transnational crime policy networks have exemplified how issues under APSS seek to operationalise a strong policy network of information and resource exchange by highlighting the proactive role that ASEAN can play in reinforcing an open, transparent, and inclusive regional architecture while maintaining its centrality through its centred role in driving initiatives and mechanisms to combat transnational crime (ASEAN Secretariat, 2009). With this, it could be further understood how networks established to cooperate over transnational crime issues boost policy networks by strengthening policymaking, decision-making and implementation phases. Using policy network analysis, the idea of the ’node-to-node relationship’ ( Caballero-Anthony, 2014) (also understood as actors-to-actors interaction) and how it encapsulates ASEAN’s centrality and the central position of ASEAN in the network is exemplified through ARF. It is an essential working concept for APSS and the transnational crime policy network(s).

In the AMMTC policy networks, engagement with NSAs in addressing counterterrorism issues and implementing ASEAN plans emphasised the importance of developing strong ties and positive engagement with ASEAN external parties ( ASEAN Secretariat, 2017). A distinct difference between transnational crime and policy networks related to territorial disputes and traditional security issues is the openness underlying the patterns of interactions and flow of resources. It also highlights that the interests and will of member states are key factors influencing policy outcomes.

Given the sensitive nature of the APSS, policies concerning the maintenance of security are approached with caution, guided by the ASEAN principles of sovereignty and non-interference. This could lead to a standstill or implementation gaps in policymaking. However, the transnational crime policy network presents a contrasting picture, characterised by high dynamism and the active involvement of NSAs in non-traditional security-related issues. This highlights the significant role member states (state actors) play in shaping the policy network, underscoring their autonomy within ASEAN. Consequently, NG assumes vital importance in ASEAN as it allows for a better understanding of the policy network’s nature and role and the overall policy subsystem. It employs the concept of centrality and resource dependence to depict the dynamics of the policy network. Despite varying characteristics, ASEAN’s norms and principles can influence policymaking if member states are willing to align with them.

## Discussion

The article employed the lens of NG to analyse the case studies from the AEPS and APSS. By examining AFTA and ASW in the AEPS and the ARF and AMMTC policy networks in the APSS, insights are gained into the dynamics of policy networks, member states’ motivations, and the role of ASEAN’s centrality in policymaking.

In the case of AFTA, traditional indicators such as the share of intra-regional trade failed to capture the true purpose and achievements of ASEAN’s economic integration. Mainstream theories of regional economic integration focused solely on economic gains, but in reality, AFTA served as a catalyst for ASEAN’s participation in the liberalised global economic order and its quest for enhanced centrality in regional institutional arrangements. Despite the lack of growth in intra-ASEAN trade, it was partly a sign of success, as it reflected ASEAN’s tariff liberalisation strategy and the pursuit of globalisation. In this case, AFTA has brought the region closer through economic ties where member states can be recognised for playing an active role in establishing the AFTA policy network. Therefore, it is crucial to understand the ASEAN context regarding economic integration and map it to what the AFTA aims to achieve. This highlights the importance of going beyond traditional quantitative indicators of intra-ASEAN trade share to total trade and understanding the needs of member states in pursuing multilateralism.

Similarly, the analysis of the ASW in the AEPS showcased the dynamics of NG, where customs cooperation and trade facilitation were achieved through collaboration among state actors within the policy network. The ASW’s implementation demonstrated the potential for enhancing intra-ASEAN trade and improving the region’s competitiveness. However, it was observed that the adoption of the ASW varied among member states, reflecting the heterogeneity of their interests within the policy network. This highlights the need to consider diverse motivations and interests when examining policy networks within ASEAN.

Shifting to the APSS, the case of the ARF exemplified NG through its structured set of autonomous entities engaged in persistent interactions. As a platform for dialogue and consultation on political and security issues, the ARF enabled member states and dialogue partners to meet, promote their interests, and extend their presence in the Asia-Pacific region. However, the effectiveness of the ARF was limited by the member states’ commitment to node-to-node interactions. The success of the ARF in fostering confidence-building and preventive diplomacy depended on the willingness of member states to engage and pursue cooperation actively.

In the context of AMMTC, ASEAN’s coordinated approach aimed to combat various forms of criminal activities, including drug trafficking, terrorism, and cybercrimes. The policy network involved not only member states but also local authorities, international organisations, and NSAs. The inclusive nature of the transnational crime policy network illustrated the importance of collaboration and information exchange in addressing complex security challenges. Unlike traditional security concerns, transnational crime exhibited a more dynamic policy network due to actors’ openness and willingness to exchange resources and information.

The strengths of this study lie in its application of NG to analyse the ASEAN policy subsystems. By adopting this theoretical framework, insights into the motivations of member states, the role of ASEAN centrality, and the dynamics of policy networks within the AEPS and APSS could be discerned. However, there are certain limitations to consider. Firstly, the study focused on only a few case studies within the AEPS and APSS, which limits the generalisability of the findings to other policy areas within ASEAN. Secondly, the analysis relied heavily on qualitative data, and while this allowed for a rich understanding of the dynamics, it may be beneficial to incorporate quantitative data and statistical analysis in future research. Additionally, the study did not extensively explore the role of NSAs and civil society organisations in the policy subsystems, which could be an area for further investigation.

Future research should consider the following directions further to advance the understanding of ASEAN’s policy subsystems. Firstly, expanding the analysis to other policy areas within the policy subsystem of the ASEAN Community, including the ASEAN Socio Cultural Community, would provide a more comprehensive understanding of the dynamics and outcomes of policy networks in ASEAN. For instance, while the ARF and AMMTC policy networks fall under APSS, they both harbour ubiquitous dynamics where the extent to which ASEAN’s norms and principles have different reach is reflected by how NSAs are more welcome in the latter. As such, there needs to be more study of other policy networks under the same policy subsystem to assess the Community’s performance further. Secondly, incorporating quantitative data and statistical analysis would strengthen the empirical basis of the findings and allow for more robust comparisons and generalisations. Thirdly, exploring the role of NSAs and civil society organisations in ASEAN’s policy networks would contribute to a more inclusive understanding of regional governance structures and processes and, thereby, ASEAN policymaking and performance. Lastly, in light of the pressing imperatives confronting ASEAN at present, such as the shift towards a sustainable green and circular economy and the rapid pace of digital transformation, alongside the complex geopolitical landscape and fragmentation of the global economy, which has substantial implications for policy networks and governance, it is imperative to pinpoint specific issue-areas of concern within these imperatives. This entails thoroughly examining individual issue-areas for the ‘interests’ and ‘will’ of each ASEAN member state and their ‘resource dependency’ on the Association to assess potential pathways and obstacles in policymaking and gaps in implementation within the ASEAN community-building agenda. The comprehensive analysis will assist ASEAN in determining its likely responses and delineate focus areas for effective governance and implementation under the identified policy networks in the three sectoral communities (policy subsystems) of the ASEAN Community (policy universe).

## Conclusion

The article studied ASEAN as NG concerning its regionalism, action, and performance, based on specific issue-areas considering its institutional design and processes. It exhibited the significance of the norms and principles of ASEAN and their substantive weight in policymaking within the three ASEAN communities. It highlighted that the interplay between the interests/will of the member states and the dynamics of stakeholders in a policy network offered a means to assess the functioning of both policy networks and policy subsystems in ASEAN. By establishing another framework, ASEAN could recognise the value that the utilisation of NG could offer in assessing its current performance or non-performance. The NG framework would also provide a channel to engage NSAs and evaluate their value in specific regional integration initiatives.

Significantly, the article could contribute to ASEAN’s understanding of its own regionalism, the present policymaking process, and institutional structures to address the implementation gaps and support its community-building agenda. It would also provide ASEAN officials, the ASEAN Secretariat, businesses, academics, and other stakeholders an alternative lens to gauge the Association’s responses, actions and performance specific to the issue-areas identified in the various policy networks under the three sectoral communities of the ASEAN Community.

## Data Availability

Dryad: ASEAN as network governance: an alternative lens to evaluate policymaking and performance. https://doi.org/10.5061/dryad.g1jwstqwp ( Sundram, 2023a). The project contains the following underlying data:
• Dataset – An alternative lens to evaluate policymaking and performance P.Sundram.xlsx. (All underlying sources, including in-text citations and sources with links, case studies and information/data sources, keywords and public sources, and accessible locations of the data). Data are available under the terms of the Creative Commons Zero “No rights reserved” data waiver (CC0 1.0 Public domain dedication). Zenodo: ASEAN as Network Governance: An Alternative Lens to Evaluate Policymaking and Performance. https://doi.org/10.5281/zenodo.8181228 ( Sundram, 2023b). This project contains the following extended data:
• ASEAN as NG Figure 1. (JPEG and TIFF files for figure 1). Data are available under the terms of the Creative Commons Attribution 4.0 International license (CC-BY 4.0).
