# Supplementary material for: ASEAN as network governance: An alternative lens to evaluate policymaking and performance
Source: F1000Res. 2023 Oct 2;12:1073. Originally published 2023 Aug 31. [Version 2] doi: 10.12688/f1000research.136338.2 (PMC10556558; doi:10.12688/f1000research.136338.2)
Supplement: Peer review history

# Reviewer report: Reviewer response for version 2

**DOI:** 10.5256/f1000research.156719.r211355

**Published:** 2023-10-05

**Authors:** Illango Karuppannan

**Affiliations:** 1 International Institute of Public Policy and Management (INPUMA), University of Malaya, Kuala Lumpur, Federal Territory of Kuala Lumpur, Malaysia

I have read the revised text and I approve it.

Is the work clearly and accurately presented and does it cite the current literature?

Yes

If applicable, is the statistical analysis and its interpretation appropriate?

I cannot comment. A qualified statistician is required.

Are all the source data underlying the results available to ensure full reproducibility?

Yes

Is the study design appropriate and is the work technically sound?

Yes

Are the conclusions drawn adequately supported by the results?

Yes

Are sufficient details of methods and analysis provided to allow replication by others?

Yes

Reviewer Expertise:

NA

I confirm that I have read this submission and believe that I have an appropriate level of expertise to confirm that it is of an acceptable scientific standard.

# Reviewer report: Reviewer response for version 1

**DOI:** 10.5256/f1000research.149475.r205200

**Published:** 2023-09-25

**Authors:** Aladdin Rillo

**Affiliations:** 1 Economic Research Institute for ASEAN and East Asia, Jakarta, Special Capital Region of Jakarta, Indonesia

The article takes an innovative approach by applying the Network Governance framework to ASEAN policy subsystems. This offers a fresh perspective on regional integration and policymaking dynamics, going beyond traditional indicators.  The use of the case studies is also appropriate as they provide application of the NG in different policy contexts, thus enhancing the article’s overall credibility.  Recognizing the intricacies of policy making in ASEAN, the article also highlights the need to look at ASEAN policy making as one that is shaped by each country’s development needs as well as the interaction of various stakeholders, particularly non-state actors. 

 The article's emphasis on the Network Governance framework offers a unique perspective that diverges from traditional approaches to assessing regional integration. This framework provides a more nuanced understanding of ASEAN's policymaking processes by recognizing the importance of collaborative networks and the interplay of member states' interests. This different perspective is instrumental in enhancing ASEAN's performance by facilitating informed decision-making, promoting inclusivity, and optimizing resource allocation.

 While the article provides valuable suggestions for future research, including expanding the analysis to other policy areas, incorporating quantitative data, and further exploring the role of NSAs, it will be good if the article provides forward-thinking approach to understanding ASEAN's policy subsystems by analyzing the implications of the future agenda of regional integration (e.g., ASEAN’s post-2025 vision) as well as the emerging new trends and challenges (e.g., issues on twin transition such as green/circular economy and digital transformation).  Since the issues facing ASEAN in the future are expected to be more entrenched with global economy (e.g., impact of intensifying geo-political tensions and economic fragmentation), will the NG framework still remain robust or will it be expanded to include a more structural analysis of governance issues in the region.  It will be interesting how the framework can elaborate these changes.

 Nonetheless, based on the strengths identified and the unique perspective offered by the Network Governance framework, I recommend approving this article for publication. The article's contribution to a deeper understanding of ASEAN's regionalism, policymaking processes, and institutional structures is substantial. Furthermore, the insights gained from this study could enhance ASEAN's performance in achieving its regional integration goals.

Is the work clearly and accurately presented and does it cite the current literature?

Yes

If applicable, is the statistical analysis and its interpretation appropriate?

Not applicable

Are all the source data underlying the results available to ensure full reproducibility?

Yes

Is the study design appropriate and is the work technically sound?

Yes

Are the conclusions drawn adequately supported by the results?

Yes

Are sufficient details of methods and analysis provided to allow replication by others?

Yes

Reviewer Expertise:

Trade integration, financial integration, regionalism, regional economy

I confirm that I have read this submission and believe that I have an appropriate level of expertise to confirm that it is of an acceptable scientific standard.

# Reviewer report: Reviewer response for version 1

**DOI:** 10.5256/f1000research.149475.r203215

**Published:** 2023-09-08

**Authors:** Illango Karuppannan

**Affiliations:** 1 International Institute of Public Policy and Management (INPUMA), University of Malaya, Kuala Lumpur, Federal Territory of Kuala Lumpur, Malaysia

I truly enjoyed reading this article. Although it is rather complex it makes a significant contribution to the field of regionalism. The focus on ASEAN enriches scholarship on regionalism, a field that is heavily influenced by EU and often regarded as the gold standard of regionalism.

 The author's extensive experience and knowledge of ASEAN's inner workings, as a professional staff in the ASEAN Secretariat comes clearly through this article.  

 The article presents a strong argument in support of the concept of Network Governance (NG) as a theoretical framework to appreciate why ASEAN regionalism has proceeded the way it has. The choice of the cases studies within the ASEAN Economic Policy Subsystem (AEPS) and ASEAN Political Security Policy Subsystem (APSS) are well chosen for this purpose.   

 The article also convincingly argues that broader goals and dynamics within the region should also be taken into account in in assessing ASEAN's economic integration rather than limiting to the traditional quantitative indicators, such as the share of intra-regional trade.

 However, I believe the article could benefit from a more elaborate explanation of how Network Governance (NG) can practically be employed as a tool to plan future policies and initiatives, as suggested. Clarifying this aspect would add practical relevance to the theoretical framework and enhance the article's overall utility. The author should provide explanation how this can be done. This would enhance the utility of the paper. If not, that statement should be deleted and it would not affect the rest of the paper.

Is the work clearly and accurately presented and does it cite the current literature?

Yes

If applicable, is the statistical analysis and its interpretation appropriate?

I cannot comment. A qualified statistician is required.

Are all the source data underlying the results available to ensure full reproducibility?

Yes

Is the study design appropriate and is the work technically sound?

Yes

Are the conclusions drawn adequately supported by the results?

Yes

Are sufficient details of methods and analysis provided to allow replication by others?

Yes

Reviewer Expertise:

International relations with an interest in ASEAN regionalism.

I confirm that I have read this submission and believe that I have an appropriate level of expertise to confirm that it is of an acceptable scientific standard, however I have significant reservations, as outlined above.

# Response

**Published:** 2023-09-08

**Authors:** Pushpanathan Sundram

**Affiliations:** School of Public Policy, Chiang Mai University, Chiang Mai, Chiang Mai, Thailand

Thank you for the valuable comments and suggestions. As suggested, I will remove the statement on the applicability part, which appears in the conclusion. This suggestion could be better taken up in a separate article, which I could consider next. My main focus for this article is to show how NG can work for ASEAN in the first instance. With this understanding, perhaps the reviewer could kindly consider approving the manuscript.
